# Mitral valve repair based on intraoperative objective measurement

**DOI:** 10.1038/s41598-019-41173-6

**Published:** 2019-03-18

**Authors:** Daniel Grinberg, Minh-Quyen Le, Young Joon Kwon, Miguel A. Fernandez, David Audigier, Florent Ganet, Jean-Fabien Capsal, Jean François Obadia, Pierre-Jean Cottinet

**Affiliations:** 1 Department of adult cardiac surgery, Hopital cardiologique Louis Pradel – LYON medical school, 28, Avenue du Doyen Lépine, 69677 CEDEX Bron, France; 2 Université Lyon, INSA-Lyon, LGEF, EA682, F-69621 Villeurbanne, France; 3 Department of cardiovascular surgery at Mount Sinai Hospital, Mount Sinai Health System, 1190 5th Avenue, 10029 New York City, NY USA; 4 French Institute for Research in Computer Science and Automation (INRIA), 2 Rue Simone IFF, 75012 Paris, France

## Abstract

In this paper, we propose a very innovative designed system that enables optimal length adjustment during transapical neochordae implantation for mitral valve repair, increasing accuracy and reproducibility of neochordae length adjustment. Also, such a new device allowed real-time measurement and recording of chordae tension, producing original physiological data. To the best of our knowledge, the tension of chordae had never been measured previously as precisely, especially in *in vivo* human clinical trials. Preliminary experimental data have been collected on 10 selected patients, giving us the opportunity to assess for the first time the tension applied on the chordae implanted in beating human hearts. The final goal of our measuring device is to provide reliable objective intraoperative data to improve the understanding of changes occurring after mitral valve repair (MVR). This novel measuring instrument may bring change in the paradigm of MVR by allowing repair with strong objective and quantitative, instead of qualitative anatomical analysis.

## Introduction

Cardiac valve diseases are known to be a major public-health problem^1^. Mitral regurgitation (MR) is the second most frequent valvular diseases requiring surgery in Eastern countries^2^. The regurgitation is either caused by a lesion of the valve itself (primary MR) or the consequence of a cardiomyopathy (secondary MR). The function of mitral valve (MV) chordae is to constrain the movement of mitral leaflets during systole, leading to a contact between the two mitral leaflets (coaptation phenomena), closing the left atrioventricular valve and insuring its transient sealing function. Elongation or rupture of chordae is a frequent lesion encountered in primary MR and lead to a prolapse. Mitral valve repair (MVR) with “respect” technics e.g. implantation of artificial Gore-Tex chordae (neochordae) and preserving the leaflet tissue, has shown very good short and long-term results^3^.

In conventional open-heart surgery, one of the main issues of the use of neochordae is determination of their appropriate length on an empty non-beating heart. The paradigm changed recently with introduction of novel techniques performed on beating hearts under 3D transesophageal echocardiography (TEE) control through a slight incision of the apex of the left ventricle (LV).

Neochord DS 1000 (NeoChord Inc., Minneapolis, Minnesota, USA) is currently the leader device for this application with more than 1100 implantations worldwide. This new device was designed to deploy neochordae through a transapical access in a beating heart and without cardiopulmonary bypass. It has shown promising results with high success rate, low perioperative morbidity and mortality, and good mid-term outcomes^4,5^. Such a procedure is currently quite challenging as the quality of repair can be only assessed based on the TEE guidance. Surgeons have no idea about the force they applied on the chordae.

Several works reported quantification of the tension of the chordae in *ex vivo* model. Of these works, the miniature c-shaped force transducers is the most evaluated technique^6–10^. In this *ex vivo* model, the valvular tissue is harvested *en masse* (ring, valve, and mitral sub-valvular apparatus) from an ovine or pigs. It is attached to a left ventricular simulator that has previously demonstrated its ability to simulate systolic geometry of the mitral valve, valve coaptation, and valvular regurgitation. A miniature transducer c-shaped force transducer is sutured in series in the middle of a chordae and bring information concerning chordal tension in physiological and pathologic conditions. Elliptical sensors (elliptical AFP4, Microstrain Inc. Williston, VT) could also be used to assess chordae tension by an extrapolation of measurement of transverse tension in chordae fibers. The sensor is inserted within the chordae.

Few *in vivo* beating heart studies have also been investigated using a porcine model. Tension in the artificial ePTFE neochordae was measured using a custom-made transducer with strain gauges^11,12^. To the best of our knowledge, no technology allowing measurement of neochordae tension *in vivo* in humans has been developed.

In order to enhance our understanding on physiology of MV and pathology of MR and to improve our knowledge on evolution of objective parameters occurring during mitral repair, we developed a measuring device that measures the tension applied on neochordae during transapical mitral valve repair. In a previous publication, we reported the clinical result of the use of the platform in seven patients^13^. Here we provide a full description of technical specifications of the developed device as well as the detailed step-by-step protocol of chordal tension measurement.

## Methods

### Prototype development of a new device of chordae tension measurement for clinical trials

**Figure 1:**
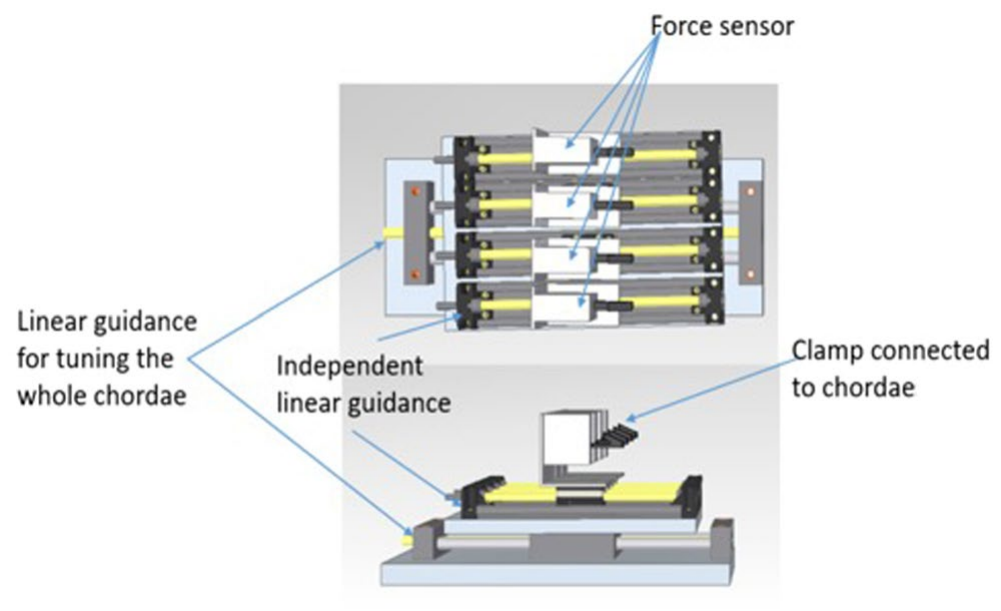
Principal scheme of chordae tension measuring device. The four force sensors are identifiable (top view) with the clamp to connect the chordae on the right side. All sensors are fixed on independent linear stage (bottom view) allowing micrometric traction of each chorda independently. Chordae can also be pulled synchronously thanks to another linear stage (bottom and left part of the device).

This study aims to determine the force generated by each chorda, which is based on high precision force sensing device. As illustrated in Fig. 1, our measuring bench consists of three principal components as follows:

- 4 identical force sensors,
- 4 metric linear translation stages (SLN-27-14 IGUS) above which fixed the sensors whose position can be independently tuned with high precision of the order of few micrometers,
- A linear stage on the bottom allowing to move the whole system (SHT-08 IGUS).

The whole system consists of four identical devices where each device is composed of a leadscrew, a force sensor (or load cell), and a clip/screw. The number of the force measuring device is chosen to be four which is considered to be standard in the case of NeoChord procedure. Each load cell is composed of a strain gage as well as a metallic elastic body which receives the force to be measured and deforms elastically by the application of this force. Indeed, through a mechanical construction, the force being sensed deforms the strain gage that enables to quantify the deformation (strain) as a change in electrical resistance, which is proportional to the measured strain and hence the applied forces. Consequently, the strain gage transducers convert the load acting on them directly into electrical signals that was implemented under Wheatstone bridge configuration for an easier treatment.

With the aim of completing the required functioning, each load cell is mounted on a leadscrew module (Drylin® product IGUS) equipped with lubricant-free linear axles that are driven by steep thread. The stroke length is selected about 100 mm with the resolution per rotation of 2 mm, making it a simple way to achieve the different levels of tension exerted on chordae with high accuracy. The main body of the guidance system is made of massive stainless aluminum. The drive was implemented via a hand wheel for an easy tuning, which was manually operated.

Accordingly, based on the above design, the developed platform allows to independently adjust the transverse tension of each chorda connected to the force device. Furthermore, the fact that the whole system fixed on the linear stage gives a possibility for surgeons to apply the same movement and as a result, the same mechanical traction to all the four chordae.

The mechanical interface between the chordae and the force sensors is performed with a crocodile clip system and a recessed screw connection, allowing them to be securely and safely attached together. As expected, the crocodile clip is installed with a pivoting link for an ease of handling and for a comfort of chordae locking procedure. The force range and the resolution of the load cell are chosen equally to ±10N and 0.01N respectively that fit well to the constraint of usual NeoChord beating heart MV implementation.

The signal is acquired using a DEWE card (SIRIUS Multi), and real-time data is processed based on the DEWE software. The data is then post-processed with MATLAB (MathWorks; Natick, MA, USA) to synchronize the measured chordae tension and the patient’s data of 3D TEE.

### Patients

Since 2016, all candidates for NeoChord procedure in our department benefit from the measurement of chordae tension during the implantations.

Our protocol received the approval of the Ethical Committee of the French Society of Cardiology. This study was performed in accordance with the ethical principles set forth in the Declaration of Helsinki. Informed consent is obtained from each patient about the complete procedure and to acknowledge that their medical records will be collected in a registry.

### NeoChord implantation and tension measurement

The procedure is conducted under general anesthesia, conventional orotracheal intubation and ventilation, without cardio-pulmonary bypass and under 2D and 3D transesophageal echocardiography (TEE) guidance. The implantation required an access to the inferior and lateral side of the left ventricle (LV) directly adjacent to the cardiac apex. This precise site is observed under 2D transthoracic echocardiography (TTE), when the patient is in the definitive operative position before the preparation. This trick help to define the optimal intercostal space for the mini-thoracotomy. Meanwhile the height and the axis of the platform is adjusted to avoid further manipulations.

Table 1 summarizes the clinical protocol, developed with engineers, anesthesiologists and surgeons. The implantation begins with the introduced of NeoChord device through the LV apex “Surgical step 1”. The prolapsed leaflet is then cached and “harpooned”. A neochordae is attached on the free-edge and exteriorized through cardiac apex (“Surgical step 2”). ePTFE sutures (GORE-TEX^®^ CV-4, W. L. Gore & Associates, Flagstaff, AZ USA) are used for this application. The number of neochordae implanted depend on the width of the prolapsed zone and three to four neochordae are usually required.

**Table 1:** Clinical Protocol for NeoChord implantation.

Description	
**Surgical Step 1:** Access of the left ventricular apex. Establishment of two apical purse string for introduction of the device and systemic heparinization,	
**Surgical Step 2:** Implantation of Neochordae (capture of the prolapsed leaflet and spear of it under 3D TEE control)	
**Surgical Step 3:** Fine adjustment of chordae length to obtain a perfect coaptation	
**Testing step 3A**	Fixation of the chordae on the platform
**Testing step 3B**	Traction of the chorda implanted in the best location until best correction
**Testing step 3C**	Traction of all chordae, one after the other, until homogeneous tension
**Testing step 3D**	Synchronous traction until optimal TEE correction
**Testing step 3E**	End of measurement and pursuing of standard protocol
Knotting at good length to fix neochordae length and location.	
**Surgical Step 4:** Heparin reversal, drainage and closing	

**Figure 2:**
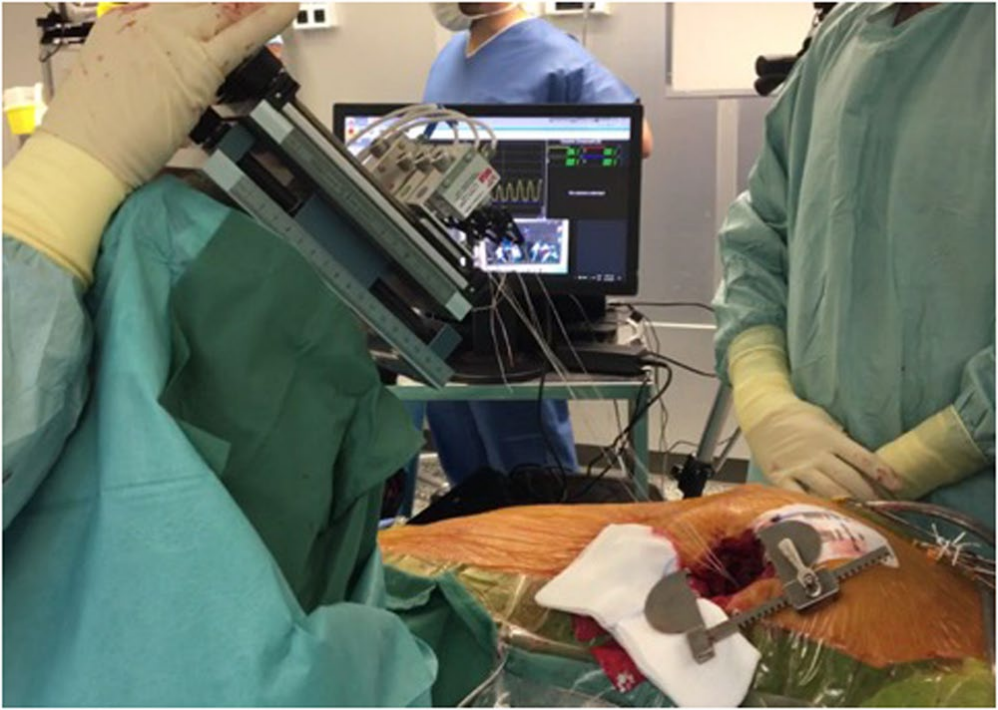
Surgical setup during the measuring phase. During this step of the protocol, the chordae were fixed to the measurement device that was brought close to the patient. While puling on the first chordae a pressure curve can be observed on the display (yellow curve).

**Figure 3:**
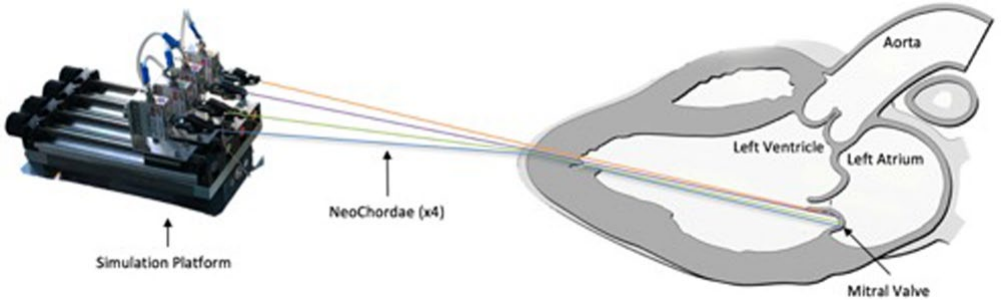
Implementation of chordae to mitral valve. In this example, four chordae are attached to the posterior leaflet of the mitral valve. Chordae are exteriorized through the apex and attached to the tension measuring device via crocodile clips.

At the end of “Surgical step 2”, three to four neochordae are thus stretched between the free-edge of the prolapsed segment and cardiac apex, and the distal part of implanted neochordae oscillates with mitral movements. The length of the neochordae has to be precisely adapted to obtain a normal coaptation to treat the regurgitation. The evaluation of the tension directly interfered at that step (“Surgical Step 3”). The device is brought closer to the patient only during the measurement phase (Fig. 2). The neochordae are connected to the measuring device through “crocodile” clips (e.g. machine-patient interface, “testing Step 3A”, Fig. 3). In the first generation of the platform, the attachment part was not sterile. The sterile sutures, however, were sufficiently long (120 mm) to allow placement of the unsterile platform far from the patient. On the other hand, in a second generation, the whole system is optimized, and the attachment system can be sterilized.

We then start continuous measurement and recording of chordae tension. Additionally, as is standard, we also collected physiological parameters that includes the blood pressure, the electrocardiogram (EKG), the surgical data based real-time cameras, and the 3D TEE. The conventional anesthesiologic protocol was followed in order to maintain a mean blood pressure between 70 and 80 mm Hg. Thus, the variation of recorded pressure only depends on the traction adjustment but not on the hemodynamic changes.

**Figure 4:**
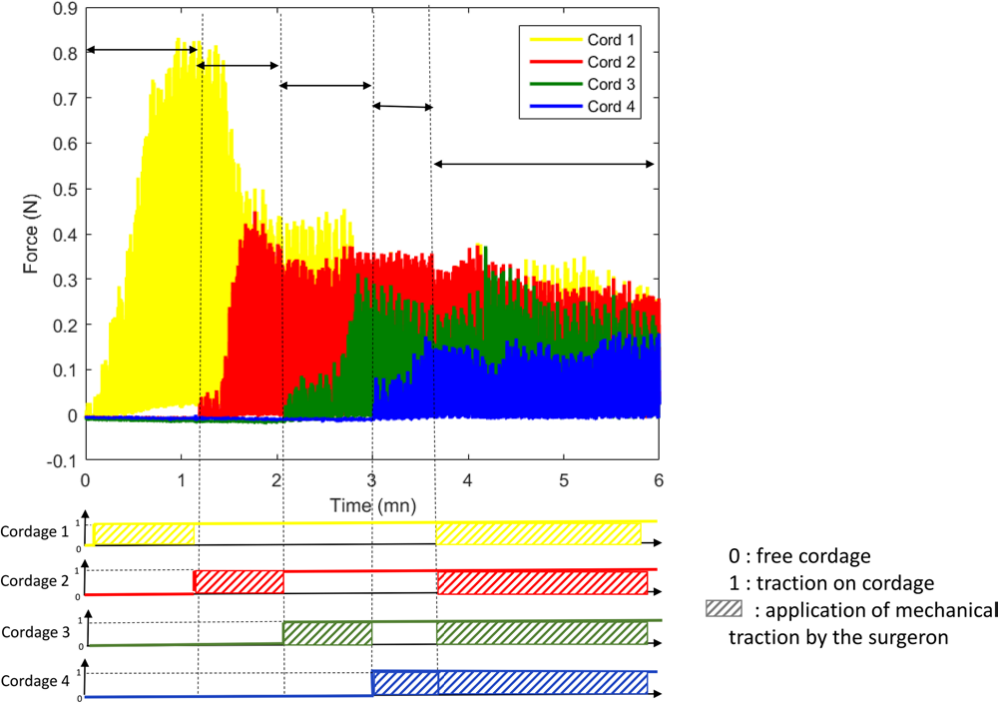
Real-time tension measurements during surgery procedure. The first phase consists of the traction of the chorda implanted in the best location (yellow) until plateau value (Testing step 3B). The tension of the first chorda reaches a plateau value of 0.8N (minute 1). Then all chordae are set in tension one after the other, until tension is homogenously spread to each chorda (Testing step 3C).

We start to apply traction on the chordae that is in the center of the flailing area thanks to a millimeter screw and under TEE control (“testing Step 3B”, Fig. 4). After obtaining a stable tension (plateau value), the other chordae are then tracked, one at a time, with an individual screw (“testing Step 3C”). When we achieve an equivalent tension on all chordae, a traction on all chordae is applied thanks to the principal screw under TEE control until obtaining a perfect coaptation (“testing Step 3D”). When the correction is optimal (good echocardiographic result, chordal tension low and equally spread on each chordae), the measurements are then stopped, and the chordae are fixed at the apex of the left ventricle at the optimal length.

The resistance of this passageway through LV apex is not quantifiable but seems insignificant.

## Results

**Figure 5:**
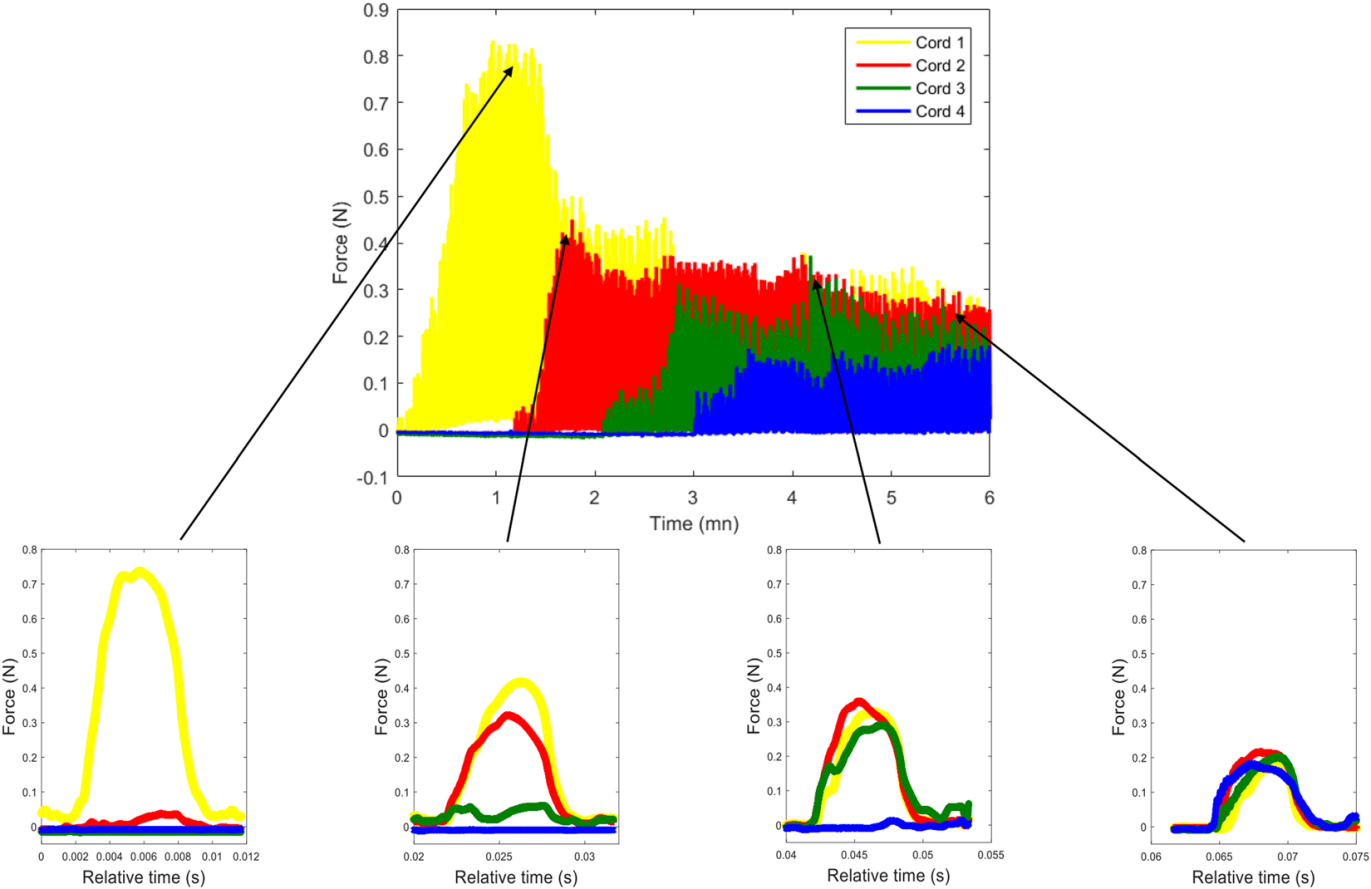
Traction on four chordae. When a first chordae is pulled the tension measured on this single chorda was find between 0.8 and 1N. This tension was then divided by the number of chordae set in tension. Thus, on the right insight we observe that when 4 chordae are set in tension, the tension applied in each chorda was homogeneous and low.

The systolic plateau value by traction on a single neochorda (the one implanted in the center of the flail area) was observed between 0, 8 to 1 Newton for all patients. When a second chordae was set in tension the force amplitude of the first chordae drastically was decreasing from 0.8N to around 0.4N and the tension of the second chordae was equal. That is, there was a reduction of forces by half when two chordae were pulled, making the sum of tension applied on the whole system unchanged. As expected in Fig. 5, similar result has been achieved when the third and the fourth chordae were active. Accordingly, setting in charge of each chordae one after the other makes the tension divided by the number of chordae, leading to good repartition of force between all the chordae. As it could be seen, a tension of 0.8N was obtained when one chorda was set whereas it fell down to 0, 2N when four chordae are in charged (Fig. 5). The result leads to an important conclusion of the fact that the tension decreased on each chorda as much as the number of chordae increased. Although this finding is intuitively obvious, we report *in vivo* physiological data for the first time in a clinical trial based on our proposed measurement platform.

As the synchronous traction of all chordae is performed until total correction of the regurgitation, the sum of tension on all chordae gradually decreases.

In case of excessive traction on the chordae, a mitral regurgitation reappears because of tethering excess and we a rising of chordal tension can be observed.

## Discussion

### What does the tension measurement teach us?

Our results concerning magnitude of measured forces are in agreement with the modeled values reported on previous publications^6–10^. If a unique chorda maintains a leaflet, the tension apply in this chorda is somewhat lower than 1 Newtons (equivalent to approximatively 100 grammes). This force is applied on human basal chordae of 1.2 to 1.3 mm thickness and on marginal chordae of 0.7 to 0.8 mm thickness. A stress–strain curves characterized by an ultimate stress values is around 35 MPa^14^, confirming the important and adequate strength of the native chordae.

It is noteworthy that our result is somewhat different with the one reported on Granier *et al*.^15^. In their study, tension on secondary chordae is measured *in vivo* with c-shaped transducer after creation of a large mitral valve prolapse (MVP) without MR using a large autologous oval pericardial patch sutured along the base of the AML. They observed a constantly decreasing in tension after MVP due to a redistribution of chordal tension. However, no further investigation has been come out after this work, according to the best of our knowledge.

This work gave us an explanation to understand natural history of MR. The tension on mitral chordae is proportional to MR level. At the initial state of dystrophic disease, occurrence of a leak lead to an increasing in tension on chordae. The phenomena lead to spontaneous and inescapable tendency towards degradation. Further analyses should be investigated to confirm these results in order to improve our knowledge concerning natural history of mitral disease.

### Interest of micrometric pulling system

According to NeoChord procedure, progressive adjustment of length of neochordae is performed manually using forceps (pulling, releasing, on chordae after the other) under TEE control until perfect correction of the mitral regurgitation^11^. Chordae are alternatively released and pulled in order to obtain an optimal morphological result. The platform allowed a synchronous or sequentially traction of chordae, bringing an easier and more precise adjustment than the conventional protocol. To the best of our knowledge, the operative time with the use of our new platform is considered to be similar with those reported in publications of reference centers^16^. Nonetheless, a deeper comparative assessment between the standard protocol and ours should be carefully investigated in the next step of this work with a bigger cohort size in order to draw firm conclusions.

### Advocacy for intraoperative assessment of objective parameters

During “testing step 3D”, a slight regurgitation could persist and minimal synchronous traction was often required to obtain a perfect TEE results. Throughout this final correction, we observed that the sum of tensions in all chordae was decreasing while increasing the traction. In other words, in a normal state, the tension applied on all chordae converges to a minimum value.

In some cases, despite resolution of regurgitation, a billowing of the posterior leaflet persisted. A slight traction on all chordae allowed a correction of the billowing with a decrease in chordae tension again. These two observations demonstrate the insufficient ability of echocardiography to assess the quality of mitral repair and the adjuvant information bring by objective intraoperative assessment.

Two safety interests of tension measurement also came from this work.

Firstly, among all the elements set in tension during chordae traction, the most fragile one is the leaflet. In case of excessive traction, the valvular tissue will then shred before the Gore-Tex. The number of tensions is thus providing a safety aspect preventing an excessive traction and potential leaflet lesions.

Secondly, after several implantation with tension measurement, aberrant tensions were detected despite good morphological results. These data warned us on surgical impairment like inappropriate positioning, winding of artificial chordae, winding of artificial and natural chordae, or insufficient number of neochordae implanted. In these cases, TEE was not contributive and objective assessment led to optimized repairs.

Last published data concerning transapical neochordae implantations with standard protocol (without tension measurement) reported an actual incidence of 1-year-patient failure of 11% despite initial good outcomes^16^. Serval reasons have been emphasized to explain these results such as long length of chordae, non-physiological length, an absence of associate annuloplasty.

We can assume that novel measurement devices could bring changes in paradigm while providing tools that allow repair based on strong objective analysis instead of subjective anatomical analysis based conventional procedures. Pursuing of intra-operative measurement and long-term analysis will be useful to confirm this hypothesis.

## Conclusion

This work proposed an innovative device for physical, real-time real-time data acquisition about MR pathology and its repair. The results of our initial experience based on chordae tension measurement are encouraging and are in line with most of the previously published works. Several physiological notions intuitively obvious are now confirmed for the first time thanks to our *in vivo* clinical testing (homogeneous repartition of subvalvular tension on all chordae, minimum stress of the subvalvular apparatus when the correction is optimal).

Factors determining the long-term durability after a plasty are numerous (e.g. LV remodeling, annular stability), and long-term follow-up and a greater number of included patients are needed to assess the prognostic value of this new parameter.

Tension measurement during a NeoChord procedure could prevent inappropriate correction resulting in aberrant tension and prevent leaflet tearing caused by excessive traction during adjustment phase.

We already considering generalizing its use for all transapical neochordae implantation. This requires several technological optimizations in order to fulfill the strict requirement specifications inherent to a commercial use.
